# Navigating the Diagnostic Maze: A Case of Post-COVID-19 Tenosynovitis

**DOI:** 10.7759/cureus.73849

**Published:** 2024-11-17

**Authors:** Kasture Puspalingam, Malarkodi Suppamutharwyam

**Affiliations:** 1 Medical, Selayang General Hospital, Kuala Lumpur, MYS; 2 Geriatrics, Selayang General Hospital, Kuala Lumpur, MYS

**Keywords:** covid 19, musculoskeletal symptoms, quality of life, tenosynovitis, unvaccinated

## Abstract

Post-COVID-19 sequelae can include various complications, including musculoskeletal manifestations. Unvaccinated against COVID-19, a 21-year-old woman initially experienced severe COVID-19 and subsequently recovered. She later developed mild COVID-19, which was followed by severe myalgia and joint pain in her upper extremities. After a third COVID-19 infection, she experienced intense pain in multiple hand joints and lethargy. The blood tests were normal, and the ultrasound report revealed tenosynovitis of the wrist joint. Her pain improved with combined treatment approaches.

To our knowledge, this is the first case report of post-COVID-19 tenosynovitis. We have also highlighted the predisposing factors for the development of tenosynovitis, which may include being unvaccinated and lifestyle changes, such as a sedentary routine. This case also emphasizes the need for further research into the long-term effects of the condition on mobility and quality of life.

## Introduction

The world has seen an explosive spread of severe acute respiratory syndrome-coronavirus-2 (SARS-CoV-2), leading to the emergence of a new respiratory pandemic known as coronavirus disease 2019 (COVID-19). Although patients with COVID-19 can recover completely, several individuals experience persistent symptoms after the acute phase of the infection and may develop a broad spectrum of sequelae [1]. Beyond pulmonary complications, frequent COVID-19 sequelae include cardiovascular, neurological, or hematological manifestations.

Unquestionably, musculoskeletal symptoms are also reported in a significant proportion of post-COVID-19 patients [2]. A study by Bakilan et al. found that 72% of patients with post-acute COVID-19 reported fatigue, and 61% and 44% of patients, respectively, reported myalgias and arthralgias [3]. In addition to that, a study by Solehan HM et al. states that lethargy, generalized weakness, and myalgias were the most common post-COVID-19 symptoms among Malaysians [4]. 

It has caused a substantial burden on healthcare systems. These sequelae may result in a reduced quality of life, increased dependency, and difficulties with activities of daily living.

Following the global pandemic, Ciaffi, J et al. conducted a systemic review highlighting new-onset rheumatic diseases such as rheumatoid arthritis, polymyalgia rheumatica, and axial spondyloarthritis, developing in association with COVID-19 [5]. Therefore, there should be an established algorithm for managing cases such as patients presenting with musculoskeletal symptoms such as arthralgia, lethargy, and myalgia, particularly in young women, where autoimmune conditions must be excluded.

Clinicians are frequently confronted with the dilemma of balancing thorough patient evaluation with resource constraints when managing these cases. Our case report contributes to the growing body of literature by systematically excluding infectious, inflammatory, and autoimmune etiologies as potential causes of post-COVID-19 tenosynovitis. We propose that comprehensive clinical evaluation, standard biochemical markers, and imaging are typically sufficient for diagnosing post-COVID-19 tenosynovitis. We have also reported on the predisposing factors for the development of the condition.

## Case presentation

A 21-year-old woman with no significant past medical history presented with polyarthralgia. She reported three previous COVID-19 infections within the past two years, including a severe case in August 2021 requiring high-flow oxygen therapy. The patient never received the COVID-19 vaccine.

The second COVID-19 infection occurred a year later, for which she was under home quarantine and remained stable throughout the isolation period. However, a week after the home quarantine, she experienced migratory joint pain and swelling involving left and right wrist joints. She was seen by a private healthcare practitioner and was prescribed paracetamol. However, while there was improvement in pain and joint swelling, complete resolution did not occur. Consequently, she was absent from work for several days.

There was no history of morning stiffness, back pain, conjunctivitis, vaginal discharge, weight loss, anorexia, rash, nail changes, or inflammatory bowel disease. Family history was negative for psoriasis, inflammatory bowel disease, and spondyloarthritis. The patient denied any history of connective tissue disease, illicit drug use, or alcohol abuse.

Despite regular analgesia with paracetamol and non-steroidal anti-inflammatory drugs (NSAIDs), the patient's pain persisted at a severe level (8/10). The persistent pain contributed to anxiety, insomnia, and low mood.

On admission, vital signs were within normal limits: blood pressure 124/85 mmHg, heart rate 86 bpm, respiratory rate 22 breaths/min, afebrile, and oxygen saturation 98% on room air. The patient appeared well without evidence of sepsis, skin rash, or conjunctivitis. Cardiovascular and respiratory systems were unremarkable on examination.

A left wrist examination revealed tenderness and swelling, primarily involving the distal interphalangeal (DIP) joint, along with a reduction in range of motion. No overlying skin, nail changes, or sensory abnormalities were noted.

Blood investigations, including autoimmune and rheumatological panels, were unremarkable (Table 1).

**Table 1:** Blood investigations

Laboratory parameters	Patient’s results	Reference ranges
Hemoglobin (HGB)	14.4	140-180 g/dL
White blood cells (WBC)	11.52	3.5-10.5 10^9^/L
Platelets (PLT)	400	140-400 10^9^/L
Erythrocyte sedimentation rate (ESR)	46	4-20 mm/h
Potassium (К)	4.1	3.5-5.6 mmol/L
Sodium (Na)	137	136-146 mmol/L
Urea	2.9	3.2-8.2 mmol/L
Creatinine	80	74-134 µmol/L
Total protein	82	66-83 g/L
Alanine aminotransferase (ALT)	17	0-50 U/L
Aspartate aminotransferase (AST)	22	0-50 U/L
C-reactive protein (CRP)	0.5	0-0.5 mg/dL
Activated partial thromboplastin time (APTT)	42	32-42 seconds
International normalized ratio (INR)	1.1	0.7-1.1
Prothrombin time	12.4	11-15.3 seconds
Complement 3 (C3)	1.64	0.70 - 1.65 g/L
Complement 4 (C4)	0.45	0.16 - 0.54
Immunoglobulin G (Ig G)	8.75	6.0 - 16.0g/L
Immunoglobulin M (Ig M)	0.64	0.4 - 2.5g/L
Immunoglobulin A (Ig A)	1.56	0.8 - 3.0g/L
Rheumatoid factor (RF)	Negative	
Anti-double-stranded DNA (Anti-dsDNA)	Negative	
Antinuclear antibody (ANA)	Negative	
Anti-smooth muscle antibody	Negative	
Anti-liver-kidney microsomal (anti-LKM) antibodies	Negative	
Extractable nuclear antigen (ENA)	Negative	
Hepatitis B	Non-reactive	
Hepatitis C	Non-reactive	
Human immunodeficiency virus (HIV)	Non-reactive	

A plain radiograph (X-ray) of the left wrist was unremarkable. The patient's absence of septic features, normal inflammatory markers, and negative autoimmune screen prompted an ultrasound of the affected joints. Ultrasound of the left hand revealed thickening of the extensor carpi radialis longus and brevis tendons to 4.8mm (normal range in women: 3-4mm). The radiocarpal, midcarpal, and distal radioulnar joints appeared normal with no evidence of effusion or synovial thickening. The scapholunate ligament was intact, and no ganglion cysts were identified. The Guyon canal was unremarkable.

Based on the patient's clinical presentation, negative inflammatory markers, and ultrasound findings suggestive of tenosynovitis, a diagnosis of post-COVID-19 sequelae manifesting as tenosynovitis was considered. Following consultation with the rheumatology and orthopedic teams, treatment commenced with nonsteroidal anti-inflammatory drugs. Subsequent rehabilitation focused on restoring function and independence. Within two weeks, the patient achieved significant improvement, with resumption of activities of daily living and eventual return to work. Concomitantly, the initial low mood and anxiety symptoms resolved as pain and physical function improved. 

## Discussion

COVID-19 itself is primarily known for affecting the respiratory system, but there is evidence that the virus can also have a range of systemic effects, including impacts on the musculoskeletal system. Tenosynovitis is an inflammation of the lining of the sheath that surrounds a tendon. As regards the correlation between COVID-19 and tenosynovitis, we hypothesize that both direct and indirect effects of the viral disease may contribute to the pathogenesis of it.

Cellular penetration of SARS-CoV-2 occurs via the angiotensin-converting enzyme 2 (ACE2) receptor and the serine protease, transmembrane protease serine 2 (TMPRSS2) [6]. With regard to human skeletal muscle tissue, many cells express ACE2 and TMPRSS2, including smooth muscle cells, endothelial cells, synovium, fibroblasts, and monocytes. Although SARS-CoV-2 has not been specifically detected in tendons and ligaments, it is suggested that the synovium is a potential site of direct virus infection [7] causing the inflammation.

COVID-19 not only directly infects cells outside the lungs but also causes indirect effects due to the body's response to the virus. These effects are linked to a cytokine storm and widespread inflammation, which can affect many organs, including musculoskeletal tissues [8]. We postulate that the inflammatory cascade causes tenosynovitis.

It should be borne in mind that, beyond the direct and indirect effects of SARS-CoV-2, the virus may also contribute to the development of tenosynovitis due to significant lifestyle changes caused by the pandemic, particularly those from quarantine. A sedentary lifestyle is linked to various negative effects, with numerous reports showing that a lack of physical activity leads to and worsens musculoskeletal pain and discomfort [9]. In our case, the patient's condition was notably debilitating, leading to multiple days of work absence and significant limitations on daily activities, which can be seen as a result of these lifestyle changes and the exacerbation of musculoskeletal symptoms.

In addition to that, a cross-sectional study in India by Madhan Jeyaraman et al. found that musculoskeletal symptoms were significantly greater in unvaccinated individuals [10]. This suggests that being unvaccinated could be a contributing factor to the severity of symptoms that our patient was experiencing.

Given the diagnostic challenges associated with post-COVID-19 tenosynovitis, clinicians must maintain a high index of suspicion when evaluating patients presenting with polyarthralgia. A systematic approach, including a comprehensive history, detailed physical examination, and targeted laboratory investigations, is essential to exclude infectious, metabolic, inflammatory, and autoimmune etiologies of joint pain and swelling.

Given the patient's age, gender, and absence of characteristic clinical or radiological features, differential diagnoses including rheumatoid arthritis, gout, and septic arthritis were considered unlikely. The absence of typical gouty manifestations, negative rheumatoid factor, and a favorable response to nonsteroidal anti-inflammatory drugs further supported this conclusion. Moreover, the lack of fever, multiple joint involvement, and elevated inflammatory markers excluded septic arthritis.

Management options for post-COVID-19 tenosynovitis typically include nonsteroidal anti-inflammatory drugs (NSAIDs). Adherence to appropriate NSAID dosing and duration is crucial to optimize pain management and prevent prolonged joint immobilization, as observed in our case. Early initiation of rehabilitation is equally important to restore function and independence.

**Figure 1:**
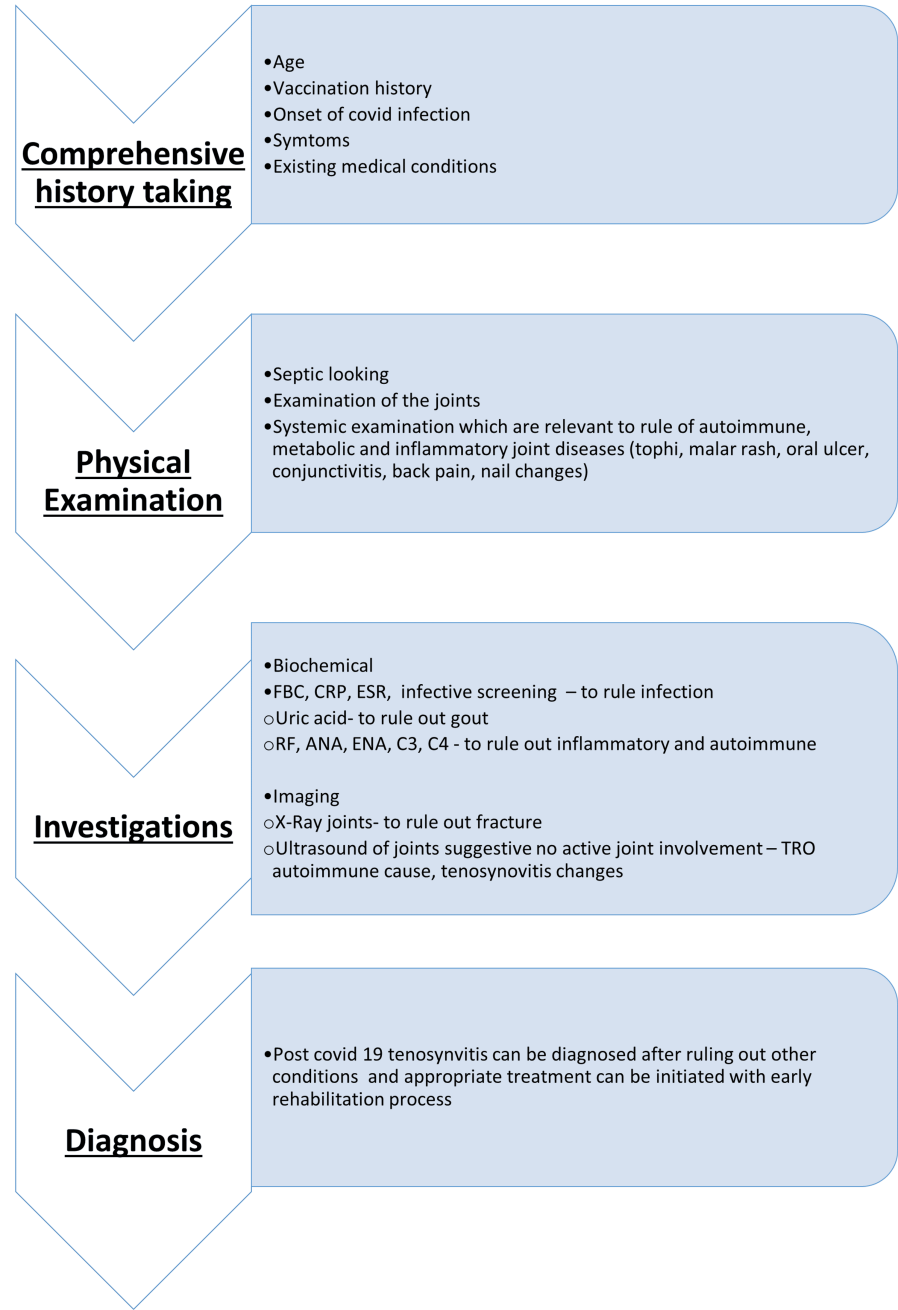
A proposed algorithm for managing patients with post-COVID-19 musculoskeletal symptoms ESR: erythrocyte sedimentation rate; FBC: full blood count; CRP: C-reactive protein; RF: rheumatoid factor; ANA: antinuclear antibody; C3: complement 3; C4: complement 4

A proposed algorithm for managing patients with post-COVID-19 musculoskeletal symptoms is presented in Figure 1.

## Conclusions

This case highlights the emerging clinical entity of post-COVID-19 tenosynovitis, emphasizing the need for a comprehensive diagnostic approach to exclude other potential etiologies. Early recognition and appropriate management are crucial to mitigate the significant impact of this condition on patient quality of life. Further research into the pathophysiology and optimal management strategies for post-COVID-19 tenosynovitis is warranted.
